# Phase-dependent closed-loop deep brain stimulation of the fornix provides bidirectional manipulation of hippocampal theta oscillations

**DOI:** 10.1016/j.brs.2025.04.019

**Published:** 2025-04-29

**Authors:** Isaac Grennan, Brook Perry, Anna Verghese, Melissa Jones, Oliver Härmson, Colin G. McNamara, Andrew Sharott

**Affiliations:** 1 Medical Research Council Brain Network Dynamics Unit, Nuffield Department of Clinical Neurosciences, University of Oxford, Oxford, United Kingdom

## Abstract

**Introduction:**

Alzheimer’s disease (AD) has very limited treatment options and therapies to prevent or reverse neurodegeneration remain elusive. Deep brain stimulation (DBS), whereby high-frequency pulses of electricity are delivered continuously to a specific part of the brain, has been trialled as an experimental treatment for AD. In AD patients, continuous, high frequency DBS targeted to the fornix (fx-DBS) has been shown to be safe, but not reliably effective across patients. In movement disorders, high-frequency DBS is thought to act as a virtual lesion, disrupting pathophysiological activity. In AD, it may be more advantageous to use stimulation to reinforce or rebuild oscillatory activities that are disrupted by the disease process. A primary candidate for such a target is the hippocampal theta oscillation, which provides a temporal framework for mnemonic processing and is altered in rodent models of AD.

**Material and methods:**

We applied closed-loop electrical stimulation to the fornix of rats traversing a linear track, triggered by different phases of the ongoing theta oscillation in the hippocampal local field potential (LFP) using the OscillTrack algorithm.

**Results:**

Stimulation at different target phases could robustly suppress or amplify the theta oscillation, and these effects were significantly larger than those caused by open-loop replay of the same stimulation pattern. Amplification of the theta oscillation could be achieved irrespective of the locomotor speed of the animal, showing that it did not result from a secondary effect of behavioural change.

**Conclusions:**

Our findings demonstrate that closed-loop fx-DBS is a viable method of modulating the amplitude of hippocampal theta oscillations that could be applied in human devices to provide a constructive intervention with the potential to boost memory circuit function in AD.

## Introduction

Deep brain stimulation (DBS) of the fornix (fx-DBS), a white matter tract connecting key memory structures in the circuit of Papez, including the hippocampus, anterior thalamus and mamillary bodies (Senova et al., 2020), has been trialled to enhance impaired cognition in a number of neurological disorders (Liu et al., 2020). fx-DBS is able to induce detailed autobiographical memories (Deeb et al., 2019; Hamani et al., 2008), restore abnormal temporal lobe glucose metabolism (Laxton et al., 2010; Smith et al., 2012), reduce the rate of hippocampal atrophy (Sankar et al., 2015) and even reduce the rate of cognitive decline in Alzheimer’s disease patients (Fontaine et al., 2013; Leoutsakos et al., 2018; Mao et al., 2018). In rodent models, fx-DBS reduced the pathological hallmarks of Alzheimer’s disease (Mann et al., 2018), such as β-amyloid plaques, and improved memory performance (Gallino et al., 2019). However, clinical trials failed to find a consistent relationship between fx-DBS and improved cognitive performance across clinical populations. For instance, while fx-DBS may benefit Alzheimer’s patients over the age of 65, it worsened symptoms in younger patients (Leoutsakos et al., 2018).

Clinical trials of fx-DBS for Alzheimer’s disease have used continuous stimulation at 130Hz, a reliable protocol for the treatment of Parkinson’s disease when delivered to the basal ganglia (Hartmann et al., 2019). Whilst some of the variance in the efficacy of fx-DBS is likely a result of the anatomical placement of the stimulation electrode (Ríos et al., 2022), it could also be the case that continuous high-frequency stimulation is not the optimal protocol for AD. Indeed, it has been hypothesised that stimulation efficacy could be enhanced if its timing was synchronised with ongoing neural activity related to memory processing (Leoutsakos et al., 2018; Mohan and Jacobs, 2024). In Parkinson’s disease, DBS at specific phases of ongoing beta oscillations in the cortico-basal ganglia circuit can amplify or suppress these activities (Holt et al., 2019; McNamara et al., 2022). However, whether a similar modulation of oscillatory activity in the circuit of Papez can be achieved with phase-locked fx-DBS has yet to be established.

A promising target activity for such an approach is the hippocampal theta oscillation, which coordinates memory-associated neural activity in humans (Herweg et al., 2020; Tesche and Karhu, 2000) and rodents (Buzsáki, 2002; Drieu et al., 2018; Drieu and Zugaro, 2019). In line with this, theta oscillations are disrupted both in patients suffering with (Hsiao et al., 2013; Ishii et al., 2017; Jafari et al., 2020; Luppi et al., 2022), and rodent models of, Alzheimer’s disease (Colom et al., 2010; Mahar et al., 2017; Mehak et al., 2022; Mondragón-Rodríguez et al., 2018; Scott et al., 2016; Siwek et al., 2015; Stoiljkovic et al., 2016; Villette et al., 2010; Wang et al., 2020). Lesions that disrupt the theta oscillation in rodents are associated with impaired memory performance (Lee et al., 2013; Luo et al., 2023; McNaughton et al., 2006; Neo et al., 2012; Shirvalkar et al., 2010; Turnbull et al., 1994). These impairments can often be rescued by continuous electrical stimulation that restores the hippocampal theta oscillation (Lee et al., 2013; Luo et al., 2023; McNaughton et al., 2006; Shirvalkar et al., 2010; Turnbull et al., 1994). In these studies, stimulation is used to entrain the hippocampal circuit to an exogenously applied theta rhythm (Lee et al., 2013; Luo et al., 2023; McNaughton et al., 2006; Shirvalkar et al., 2010; Turnbull et al., 1994), highlighting the importance of the theta oscillation in memory performance. However, we hypothesise that strengthening endogenous theta, rather than imposing the oscillation on the network, could provide a more effective interaction with circuit dynamics. Moreover, this approach may allow for even greater control over the oscillation (e.g., having the capacity to both supress and amplify the oscillation).

Supporting the benefits of interacting with the endogenous theta rhythm, optogenetic stimulation of hippocampal PV interneurons on specific phases of the theta oscillation enhanced memory performance (Siegle and Wilson, 2014). Theta-locked optogenetic stimulation of the hippocampus that mimicked phase-precession was found to increase the stability of hippocampal place fields (Aoki et al., 2023). Whether a similar phase-locking of the more clinically tractable fx-DBS to the hippocampal theta oscillation is achievable and can modulate theta-rhythmicity in the hippocampal circuit is currently unclear.

To address these questions, we delivered closed-loop fx-DBS to different phases of hippocampal theta oscillations in rats traversing a linear track. We demonstrate that phase-locked stimulation can bidirectionally modulate the power of theta oscillations, and that this modulation is dependent on closed-loop interaction. These findings suggest implementing phase-locked fx-DBS in next-generation DBS devices has the potential to facilitate more targeted enhancement of hippocampal memory processes.

## Methods

This experiment was conducted in 5 male Lister Hooded rats approximately 3 months old at the time of surgery. Rats were kept on a 12-hour light/dark cycle. Animals were initially housed in groups of 2 or 3. Rats had *ad libitum* access to water in their home cages but were food-deprived at 90-95% of their free-feeding weight during behavioural testing. Following surgical implantation of a multielectrode microdrive, animals were singly housed. All experiments were carried out in accordance with the UK Animals (Scientific Procedures) Act (1986).

### Behavioural paradigm

The behavioural apparatus consisted of a 130cm long linear track connected to two 18cm diameter semi-circular arenas at the ends of the track as described in Härmson et al., 2023 (Härmson et al., 2023). Rats were habituated to being handled and to each of the experimental environments for 7 days prior to implantation of the microdrive. To encourage running, rats were rewarded with a single 20mg sugar pellet if they ran across the linear track and nose poked into the reward port.

### Electrophysiological recordings

A driveable multielectrode implant was used to record field potentials and single unit activity in the right hippocampus (Härmson et al., 2023) with a DBS electrode targeted to the right fornix (fig. S1). Wideband data was recorded, amplified and digitised on two Intan 64-channel headstages at 20,000 Hz using an RHD USB interface board (Intan Technologies) referenced to the cerebellum. Videos of all stages were triggered frame by frame at 30Hz by the behavioural software and recorded using StreamPix 8. The position of animals in each frame of the video was then determined using DeepLabCut (Mathis et al., 2018). The electrical stimulation was delivered biphasically at 50-100 µA and lasted 200µs.

### Theta-locked fornix-DBS

Online phase tracking was performed using the OscillTrack algorithm implemented on the FPGA in the Intan RHD USB interface board (McNamara et al., 2022, https://colinmcn.github.io/OscillTrack/). This was used to control the timing of closed-loop, fx-DBS phase-locked to the theta oscillation as recorded using a LFP electrode targeted to stratum oriens of CA1 of the right hippocampus. In rodents, theta oscillations occur most prominently during locomotion (Fuhrmann et al., 2015; McFarland et al., 1975; Nuñez and Buño, 2021; Rivas et al., 1996; Whishaw and Vanderwolf, 1973). To ensure a robust theta oscillation, we thus delivered our phase-locked stimulation as animals ran back and forth on a linear track. Animals were rewarded after each lap with the delivery of a sugar pellet. Stimulation was delivered at 1 of 4 different phases in each recording day (0 degrees-peak, 90 degrees-descending, 180 degrees-trough, and 270 degrees-ascending).

On each recording day for each animal, the experiment consisted of three different stages in the linear track environment: 1) *no-fx-DBS*: theta phase tracking with no fornix stimulation, 2) *phase-locked fx-DBS*: theta phase tracking used to trigger fornix stimulation targeting one of the aforementioned phases of the oscillation, 3) *replayed-fx-DBS*: replay of the stimulation trains generated by closed-loop stimulation targeting the same phase as in 2). For example, the open-loop replay control session for closed-loop stimulation at the peak phase would be the replayed stimulation trains generated from a previous session of closed-loop targeting the peak phase. The open-loop stimulation trains therefore had the same temporal and spectral properties as a previously delivered closed-loop stimulation train, but had no systematic relationship to the phase of ongoing theta oscillations in the LFP. This allowed us to differentiate the effect of the temporal pattern of a stimulation train from the effect of the stimulation being phase-locked to the ongoing theta. Phase-locked fx-DBS was only considered to have a *closed-loop* effect on a given electrophysiological or behavioural variable if it significantly differed from both the *no*-fx-DBS *and* replayed-fx-DBS controls.

These three stages were delivered in a counter-balanced order. Electrophysiological recordings were made while rats traversed the linear track in these three stimulation stages, each lasting 15-minutes. The temporal pattern with which stimulation is delivered over each full stage will be referred to as the ‘stimulation train’. Each stimulation train is therefore 15 minutes in duration. The stimulation train can be thought of as a binary timeseries which takes the value of 1 at times where stimulation is initiated and 0 otherwise (see below).

### Statistical analysis

Power spectral densities (PSDs) were calculated for each of the 3 DBS conditions and the maximum power (Max. power), frequency (Max. Frequency) and the area under the curve (AUC) was quantified between 6-10Hz. Welch’s method was used to compute estimates of the power spectral density of the 1000Hz LFP signal using a segment length of 2^12^. Max. power was calculated as the maximum power in the PSD between 6Hz and 10Hz. The frequency that corresponded to this maximum power was the Max. Frequency. The instantaneous phase and amplitude of theta oscillations was calculated from the Hilbert transform following filtering of LFP in the range 6-10Hz using a 4th order Butterworth filter (see supplemental methods for more details).

Both PSDs and the envelope of the theta oscillations were z-scored in a similar manner. First the PSDs from all sessions were converted into dB. The power spectra for the frequencies below 100Hz from each of the 3 sessions in a recording day (phase-locked fx-DBS, replayed-fx-DBS and *no*-fx-DBS) were then concatenated together. The PSDs for each session were then z-scored relative to the mean and standard deviation of this concatenated signal. For the theta envelope, we concatenated the instantaneous amplitude from the 3 sessions that make up a recording day. The instantaneous amplitude for each session is then z-scored relative to the mean and standard deviation of the concatenated envelope.

For analysis of the stimulation trains, DBS pulse times were converted to a binary timeseries at a sampling rate of 250Hz, with a 1 indicating that stimulation onset started at a particular timepoint. The PSDs and cross correlation were then calculated using the same methodology as the LFP (see supplemental methods for more details).

Paired comparisons of phase-locked fx-DBS to *no*-fx-DBS/replayed-fx-DBS were tested using the Wilcoxon signed-rank test (a non-parametric test of medians). To align with this statistical approach, we plotted the median and standard error of the median (calculated using bootstrapping) change between phase-locked fx-DBS and *no*-fx-DBS/replayed-fx-DBS. All other data was plotted as mean +/- standard error of the mean. To compare across different phases, which were not recorded in the same day, we normalised data by subtracting the *no*-fx-DBS baseline. The Kruskal-Wallis test was then used to determine non-parametrically if data from the different phases of phase-locked DBS was from the same distribution.

## Results

### Fornix-DBS could be accurately phase-locked to the hippocampal theta oscillation

**Figure 1:**
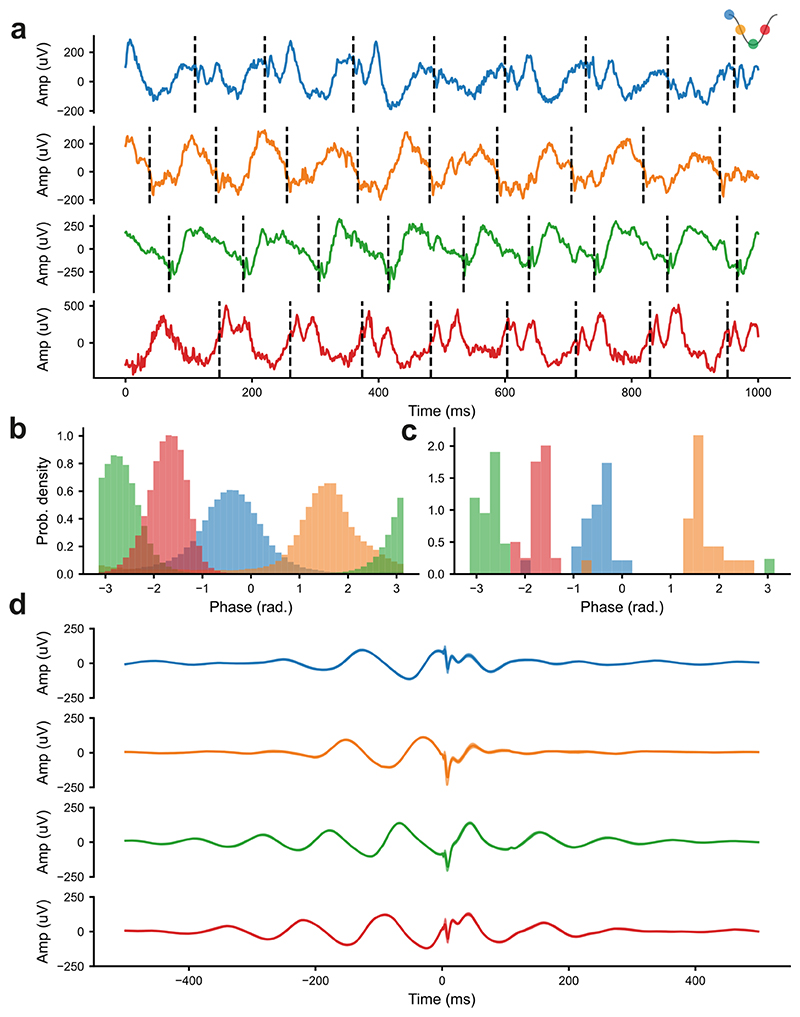
fx-DBS was reliably phase-locked to theta oscillations in HC-LFP. **a)** Wideband LFP with the stimulation artefact removed. The times where stimulation was delivered are marked with a vertical dashed line. **b and c)** The wideband LFP was filtered in the theta band (6-10Hz), and the phase that fx-DBS pulses occurred at was determined offline using the Hilbert transform. **b)** The phase of all of the DBS pulses pooled across all phase-locked fx-DBS stages for each stimulation phase. **c)** The circular mean of the phase at which fx-DBS pulses were delivered for each closed-loop stage, for each stimulation phase. **d)** The average wideband LFP triggered by fx-DBS pulses during phase-locked fx-DBS, averaged for each stimulation phase. For all figure panels, blue=peak-phase, orange=descending-phase, green=trough-phase and red=ascending-phase fx-DBS (see key in top right corner). Data presented here is from 5 rats over 83 recordings (peak-phase n=22, descending-phase n=22, trough-phase n=20, ascending-phase n=19).

In order to investigate the effects of phase-locked DBS on hippocampal theta oscillations, we first had to verify that we could deliver fx-DBS that was accurately locked to specific phases of the HC-LFP theta oscillation. Even in unfiltered raw LFP it was clear that stimulation was delivered to the target phase of the ongoing theta oscillation (Fig. 1a). To quantify the accuracy of online phase-targeting, we compared the phase at which stimulation was delivered or would have been delivered (*no*-fx-DBS), to the post-hoc Hilbert-transform based calculation of phase on the filtered signal. Without stimulation, tracking was highly-accurate, with over 75% of pulses being delivered for each target phase (Fig. S2). When stimulation was delivered there was more variability in the proportion of pulses at or close to the target phases (Fig. 1b, within a quarter cycle of the desired phase: peak-locked = 65% [80383 / 123631 stimulations], descending-locked = 72% [82125 / 114806 stimulations], trough-locked = 81% [93662 / 115311 stimulations], ascending-locked = 92% [114285 / 123823]), however a high degree of accuracy was still achieved. The circular mean of the actual phase of stimulation for each closed-loop session (Fig. 1c) and the mean stimulation-evoked potential (Fig 1d) further confirmed the accuracy of phase-targeting across the data set. There was a slight offset in actual, compared to targeted, phase when stimulation was delivered (Fig 1c, d), which thus resulted from a perturbation to the ongoing signal rather than inaccuracy in tracking. Interestingly, the degree of theta rhythmicity in the triggered averages of fx-DBS was seemingly reduced for peak and descending, relative to trough and ascending target phases (Fig. 1d).

### Phase-locked fornix-DBS bidirectionally modulated the power of hippocampal theta oscillations

**Figure 2:**
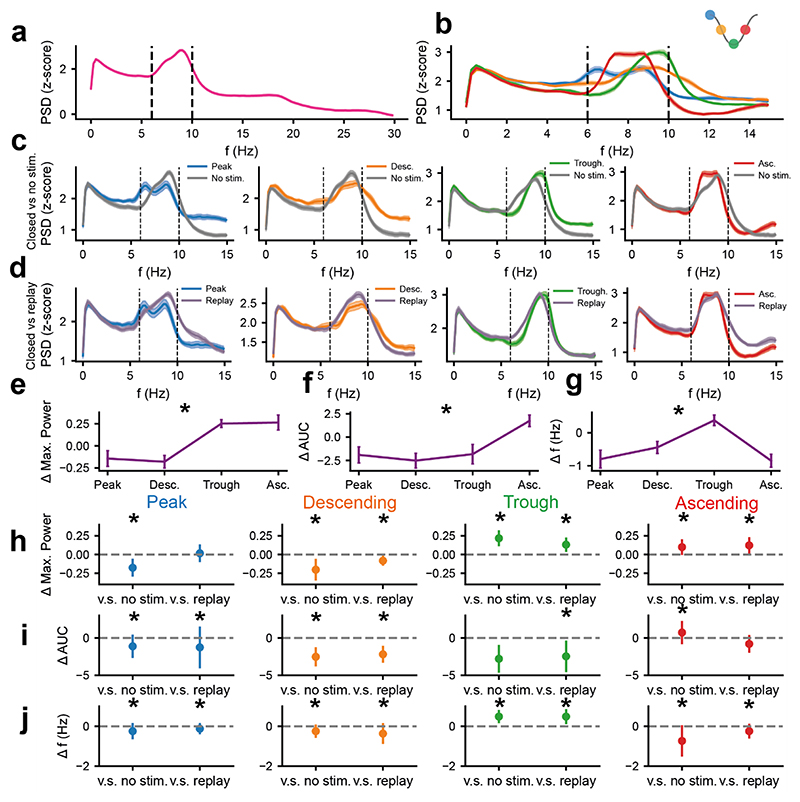
Phase-locked fx-DBS can bidirectionally modulate the power of theta oscillations. **a)** The z-scored log power spectra in the theta band in the CA1 in animals in the linear-track in the absence of fx-DBS. **b)** The z-scored log power spectra in the theta band in the CA1 during phase-locked fx-DBS, averaging across stages of each of the four stimulation phases. **c)** The z-scored log power spectra in the theta band in the CA1 during phase-locked fx-DBS, averaging across stages of each of the four stimulation phases (coloured) compared to *no*-fx-DBS (grey). **d)** The z-scored log power spectra in the theta band in the CA1 during phase-locked fx-DBS, averaging across stages of each of the four stimulation phases (coloured) compared to replayed-fx-DBS (purple). **e)** The difference in the maximum z-scored log power in the theta band (6-10Hz) during phase-locked fx-DBS relative to *no*-fx-DBS changed significantly as a function of the phase of stimulation (Kruskal-Wallis test, p<10^-5^). **f)** The difference in the area under the curve of the z-scored log power spectra in the theta band (6-10Hz) during phase-locked fx-DBS relative to *no*-fx-DBS changed significantly as a function of the phase of stimulation (Kruskal-Wallis test, p<0.0002). **g)** The difference in frequency of maximum power in the theta band (6-10Hz) during phase-locked fx-DBS relative to *no*-fx-DBS changed significantly as a function of the phase of stimulation (Kruskal-Wallis test, p<10^-4^). **h)** The change in maximum z-scored log power in the theta-band (6-10Hz) in phase-locked fx-DBS as compared to *no*-fx-DBS and replayed-fx-DBS (Wilcoxon signed-rank test, p<0.05 marked by an asterisk). Points here represent the median change in maximum log power and the error bars represent 2 x standard error of the median. **i)** The change in the area under the curve in the z-scored log power spectra in the theta-band (6-10Hz) in phase-locked fx-DBS as compared to *no*-fx-DBS and replayed-fx-DBS (Wilcoxon signed-rank test, p<0.05 marked by an asterisk). Points here represent the median change in area under the curve and the error bars represent 2 x standard error of the median. **j)** The change in frequency of maximum power in the theta-band (6-10Hz) in phase-locked fx-DBS as compared to *no*-fx-DBS and replayed-fx-DBS (paired t-test, p<0.05 marked by an asterisk). Points here represent the median change in the frequency of maximum power and the error bars represent 2 x standard error of the median. For all figure panels, blue=peak-phase, orange=descending-phase, green=trough-phase and red=ascending-phase fx-DBS (see key in top right corner). Data presented here is from 5 rats over 83 recordings (peak-phase n=22, descending-phase n=22, trough-phase n=20, ascending-phase n=19).

As others have reported in locomoting animals (Fuhrmann et al., 2015; McFarland et al., 1975; Nuñez and Buño, 2021; Rivas et al., 1996; Whishaw and Vanderwolf, 1973), theta oscillations manifested as a large peak in the power spectra of HC-LFP between 6-10Hz (Fig. 2a). A strong, stable theta oscillation was also found in the time-frequency spectra across entire recordings (Fig. S3). During closed-loop stimulation, the spectral properties of theta were clearly modulated as a function of the target phase (Fig. 2b), with both amplification and suppression in relation to *no*-*fx-DBS* (Fig. 2c) and *replayed-fx-DBS* (Fig. 2d) controls. The change in spectral power (as measured by either the maximum power or area under the curve [AUC] in the theta range) and frequency at the maximum power compared to *no*-*fx-DBS* was significantly different with respect to target phase (Fig. 2e-g). Closed-loop stimulation at the trough or ascending phase led to significant amplification of the maximum theta power (Fig. 2h), whereas stimulation at the peak and descending phase led to suppression (Fig. 2i). Shifts in the mean frequency of the maximum power of theta oscillations were observed for all phases of fx-DBS (Fig. 2j).

### The pattern of stimulation in phase-locked DBS changes as a function of target phase

**Figure 3:**
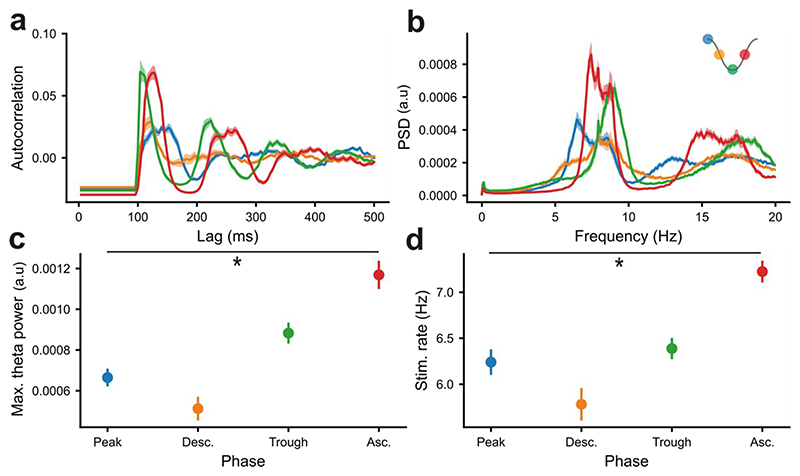
Phase-locked fx-DBS leads to different patterns of stimulation depending on target phase. **a, b)** The autocorrelation (**a**) and power spectrum (**b**) of the stimulation trains of theta-locked fx-DBS differed across the targeted phases. **c:** the maximum theta (6-10Hz) power of the stimulation train in fx-DBS targeted to the peak, descending, trough and ascending phase. The theta rhythmicity of stimulation varied significantly as a function of the phase targeted by phase-locked fx-DBS (Kruskal-Wallis test, p<10^-9^). **d:** the fx-DBS pulses per unit time for peak, descending, trough and ascending phase-targeted stimulation. The rate of stimulation varied significantly as a function of the phase targeted by phase-locked fx-DBS (Kruskal-Wallis test, p<10^-7^). For all panels of this figure, blue=peak-phase, orange=descending-phase, green=trough-phase and red=ascending-phase fx-DBS (see key in top right corner). Error bars indicate standard error of the mean.

In addition to phase-dependent changes in the LFP during closed-loop DBS, the theta-rhythmicity of the stimulation train (i.e., the pattern of stimulation delivered over a full recording stage) itself varied as a function of target phase (Fig. 3a, b). Phase-locked stimulation leads to rhythmicity in the stimulation train with a frequency around the cycle-length of the target oscillation, with amplification leading to more rhythmic stimulation due to the increased stability of the input signal and vice versa (McNamara et al, 2022). In line with this, closed-loop stimulation trains generated by the suppressing peak/descending target phases had lower theta power than the amplifying trough/ascending target phases (Fig. 3c). Overall, when the underlying hippocampal theta oscillation became stronger so did the theta rhythmicity of the stimulation pattern. In addition, more stimulation pulses per unit time were delivered with ascending-locked DBS, which was amplifying, than descending-locked DBS, which was supressing (Fig. 3d). The OscillTrack algorithm is designed such that in periods where the phase-estimate is unstable, it withholds stimulation. As the amplitude of the oscillation decreases the phase estimation will become weaker, resulting in more cycles where stimulation is withheld and a lower stimulation rate (McNamara et al., 2022). In addition, fx-DBS driven changes in the dominant frequency of the theta oscillation will also change the rate of stimulation delivery. For instance, phase-locked fx-DBS which increases the frequency of hippocampal theta-oscillations will be associated with a higher rate of stimulation as a result of locking to the shorter theta cycles.

### Phase-targeted theta amplification and suppression vary as a function of locomotor speed

The power of theta oscillations is positively correlated with the speed of locomotion (Fuhrmann et al., 2015; McFarland et al., 1975; Nuñez and Buño, 2021; Rivas et al., 1996; Whishaw and Vanderwolf, 1973). In line with this, there were clear changes in the power and frequency of theta oscillations at the transition from slow to fast movement (<20cm/s to >20cm/s Fig. S4). We next tested if phase-locked fx-DBS could modulate theta power across both fast and slow movements. To do this we calculated the mean time-frequency spectra during the transition from slow (<20cm/s) to fast movement (>20cm/s) for each stimulation condition. We then examined if there were clusters of adjacent time/frequency points where the theta-power was significantly modulated by the target phase of stimulation. To do this, we found clusters of adjacent time/frequency points where an ANOVA identified a significant (p<0.01) difference in the power of oscillations across the different target phases of stimulation. The magnitude of the f-statistic (from an ANOVA comparing the power of an oscillation at a given time-frequency pair across the different target phases) summed across all of the time/frequency points in a given cluster was significant if above the 97.5^th^ percentile of a null distribution produced by randomly permuting the phase-labels (see methods). The cluster level statistic represents how strongly the target phase of stimulation modulates the oscillatory power over the frequencies and times contained within the cluster as a whole. This approach demonstrated that the effect of stimulation was specifically restricted to the theta-band and its harmonic (Fig S4). Equally, this modulation of the theta oscillation by the target-phase was present prior to and following the speed transition. This confirms that our effects were not restricted to periods when the animal was either stationary or moving.

**Figure 4:**
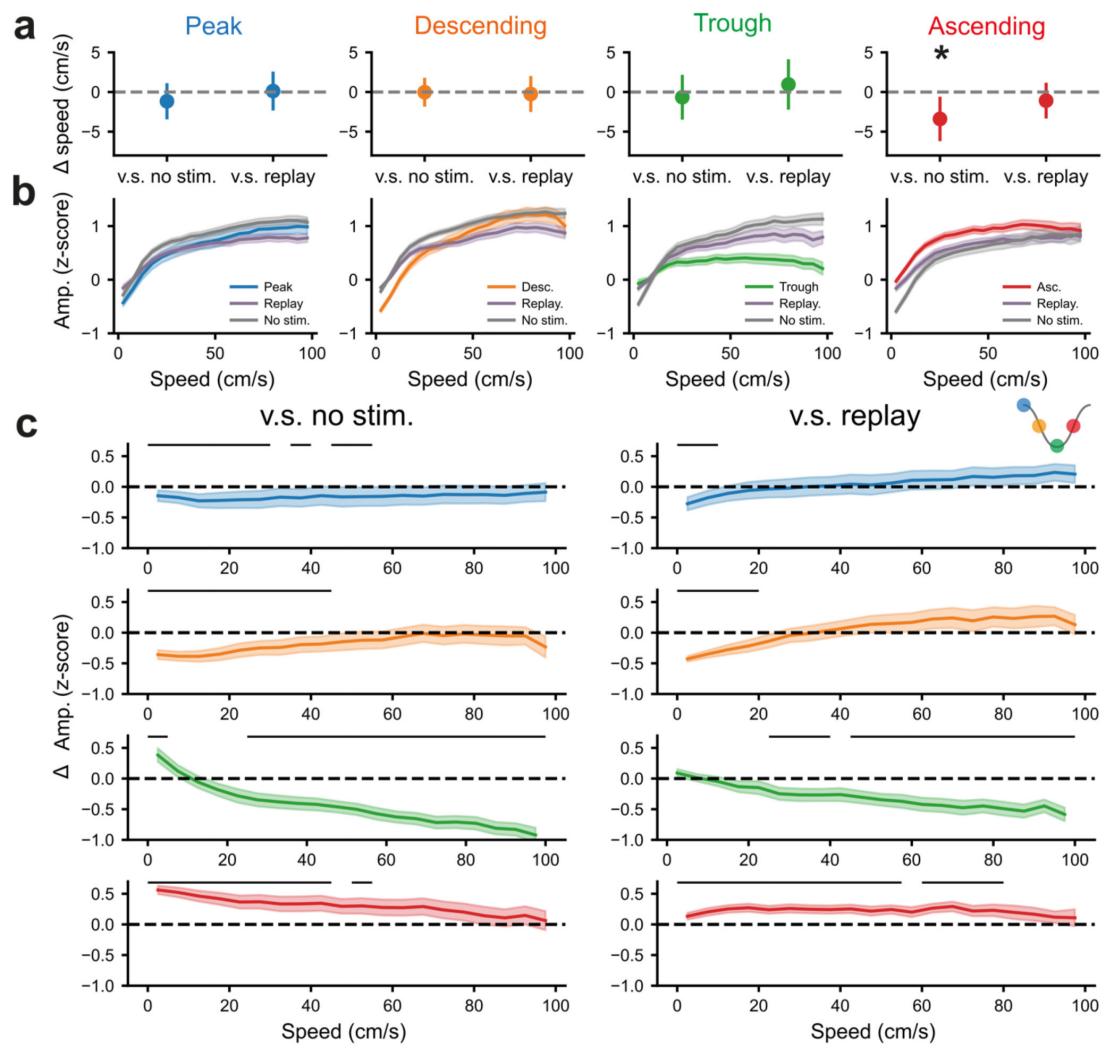
The amplitude of theta oscillations was modulated by theta-locked fx-DBS after controlling for the speed of animals. **a)** The overall speed of rats in phase-locked fx-DBS was compared to *no*-fx-DBS and replayed-fx-DBS. Points here represent the median change in speed and the error bars represent 2 x standard error of the median. Ascending-phase fx-DBS was associated with a significant reduction in running speed relative to *no*-fx-DBS (Wilcoxon signed-rank test, p=0.0056). **b)** The instantaneous amplitude of theta oscillations was calculated from the Hilbert transform of the theta-filtered (6-10Hz) LFP. This was averaged over the different speeds that rats moved at in 5cm/s bins. This allowed the amplitude of theta oscillations to be plotted as a function of the speed that animals were moving for each session. We then averaged across sessions of each of the 4 different phase conditions. The envelope of theta oscillations was plotted as a function of speed for the phase-locked fx-DBS (blue: peak, orange: descending, green: trough and red: ascending), *no*-fx-DBS (grey) and replayed-fx-DBS (purple). **c)** The difference between the theta amplitude as a function of speed during phase-locked fx-DBS and *no*-fx-DBS (left) or replayed-fx-DBS (right), periods of significance (Wilcoxon signed-rank test, p<0.05) were marked with a black bar. For all figure panels, blue=peak-phase, orange=descending-phase, green=trough-phase and red=ascending-phase fx-DBS. Data presented here is from 5 rats over 71 recordings (peak-phase n=19, descending-phase n=18, trough n=16, ascending n=18).

We next addressed whether phase-locked fx-DBS changed the relationship between theta power and locomotor speed. fx-DBS at the ascending phase, which amplified theta power when the whole session was considered (see above), reduced running speed compared to *no*-fx-DBS stages (Fig 4a). However, this speed change was not different to open-loop, replayed-fx-DBS suggesting that this was an effect of the pattern of stimulation (the most oscillatory of all the target phases [Fig. 2]), rather than a closed-loop interaction. During stimulation at the ascending phase, theta amplitude was increased across nearly all speeds as compared to replayed-fx-DBS (Fig 4b, c). Taken together, these results demonstrate that the theta modulation was not an indirect effect of change in speed or stimulation pattern. Interestingly, for peak- and descending-phase stimulation, the suppression of theta amplitude was only significant at low running speeds (Fig. 4c), demonstrating that these effects were speed-dependent. Stimulation at the trough had the opposite relationship, with significant suppression at high-running speeds as compared to replayed-fx-DBS, perhaps as a result of pushing the frequency of oscillations outside of the canonical theta band (Fig 2). Overall, these results demonstrate that the effects of closed-loop stimulation can be dependent or independent of locomotor speed as a function of target phase.

### Phase-dependent effects of open-loop stimulation

**Figure 5:**
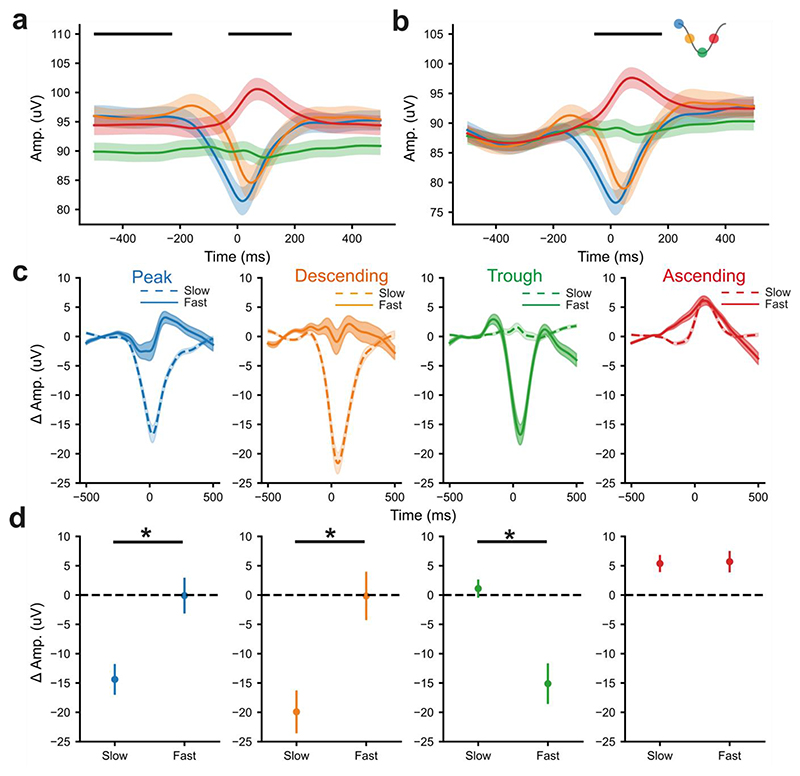
Modulation of theta amplitude by individual fx-DBS pulses was phase and speed-dependent. Replayed-fx-DBS has no systematic relationship to the phase of theta oscillations so over time each fx-DBS pulse will align with all the phases of the theta oscillation by chance. Here we relate the phase of those pulses to subsequent changes in theta amplitude. The wideband LFP was filtered in the theta band (6-10Hz). The Hilbert transform was then used to determine the phase that fx-DBS pulses occurred and the change in the instantaneous amplitude of theta oscillations. The theta cycle was divided into 4 phase conditions each pi/2 in width, centred around the peak, descending, trough and ascending phase to align with previous figures. **a)** The instantaneous amplitude of theta oscillations was triggered around replayed-fx-DBS pulses, separating by the phase of the theta oscillation that the pulse coincided with. Significant difference in amplitude between stimulus phases are marked with a black bar (Kruskal-Wallis, p<0.05, Benjamini-Hochberg procedure used to control for multiple comparisons). **b)** As in **a**, but following the exclusion of pulses with unusually high baseline amplitudes to control for baseline differences. **c**) The change in the instantaneous amplitude for each of the 4 phase bins triggered around replayed-fx-DBS, separated by whether the animal was moving slowly (<20cm/s, dashed line) or running (>20cm/s, solid line) at the time of stimulation. **d**) The mean change in amplitude in the 100ms following delivery of a fx-DBS pulse for each of the 4 phase bins separating by whether the animal was moving slowly or running. Points here represent the mean and 2 x standard error of the mean. The change in amplitude was significantly greater for slow than fast movement for peak and descending phase fx-DBS pulses, whereas the reverse was true for trough phase fx-DBS pulses (Wilcoxon signed-rank test, p<10^-10^ for all, p>0.05 for ascending phase stimulation). For all figure panels, blue=peak-phase, orange=descending-phase, green=trough-phase and red=ascending-phase (see key in top right corner). For **a** and **b**, data presented is from 5 rats over 83 recordings. For **c** and **d**, data presented is from 5 rats over 71 recordings (the subset of the 83 recordings with accompanying videos).

Whilst replayed-fx-DBS did entrain theta oscillations (Fig S5), DBS pulses spanned all phases (Fig. S5A). This allowed us to analyse the effect of a single electrical stimulation pulse on the instantaneous amplitude of the theta oscillation as a function of the phase it landed on, in the absence of closed-loop targeting. Consistent with the findings of Fig. 2, pulses falling on the peak and descending phases were associated with a decrease in instantaneous amplitude (Fig. 5a). Whereas stimulation at the ascending phase was associated with an increase in the instantaneous amplitude (Fig. 5a). This was the case even after controlling for differences in the initial baseline amplitude of the theta oscillation (Fig. 5b). Even the locomotion-phase interaction effect of DBS on theta-amplitude observed in Fig. 4 was largely replicated by single pulses of stimulation delivered in an open-loop fashion (Fig. 5c and 5d). This analysis suggests that the effects of closed-loop stimulation are due to continuous exploitation of the basic phase-dependent response of the circuits generating theta oscillations.

## Discussion

Here we demonstrate that phase-locked fx-DBS has the capacity to bidirectionally modulate the amplitude of theta oscillations. Most of our key effects were significant relative to *both no*-fx-DBS and replayed-fx-DBS. In replayed-fx-DBS, we delivered DBS pulses with the same pattern as a previous phase-locked recording on a given day. This controls for the fact that targeting different phases produced different patterns of stimulation trains, which may affect the system differently irrespective of phase-locking. Indeed, ascending phase-locked stimulation (which resulted in amplification of the theta oscillation), had greater theta rhythmicity and delivered more stimulations per unit-time than other phases of stimulation. This likely resulted from a reciprocal interaction between the phase tracking algorithm and the stimulation driven effects on the theta oscillation (McNamara et al., 2022). That is, theta-locked fx-DBS that increased the amplitude of theta oscillations increased the signal to noise ratio of the phase detected by the Oscilltrack algorithm. This in turn increased both the fidelity of phase tracking and the periodicity of the stimulation delivered by the Oscilltrack algorithm.

Much of the closed-loop suppression and amplification of the theta oscillation occurred when the animal was moving slowly. It may be easier to achieve suppression at slower speeds, where theta oscillations are weak. As the speed of locomotion of animals increases, so does the power of theta oscillations. These stronger theta oscillations may be more resilient to the suppressive effects of phase-locked fx-DBS. Equally, while present over a wider range of speeds, amplification of theta power was most readily achieved at slower speeds of locomotion. There is a physiological ceiling to the power of theta oscillations, as indicated by the plateauing increase in theta power with increasing speed across all conditions. As animals approach this ceiling, amplifying stimulation may have diminishing effects. Perhaps the most striking change in the theta-speed relationship occurs with trough-locked fx-DBS. In this instance, there was some amplification of theta power at slower speeds and a strong inhibition at higher speeds of locomotion. The net effect of this is an almost complete uncoupling of theta power from the locomotor speed of animals, although this effect may be driven by pushing the frequency of oscillations outside the canonical theta-band.

During open-loop replay, stimulation pulses were delivered across a range of different phases, allowing us to explore instantaneous phase-dependent effects without specific closed-loop targeting. This analysis showed that the phase of pulses in the replayed-fx-DBS condition predicted the change in theta amplitude caused by closed-loop stimulation at the same phase. Even the more complex effects, such as the interaction between target phase and locomotor speed, were preserved. As the replayed stimulation trains were oscillatory, they would have resulted in short runs of consecutive pulses landing at the same phase, as seen when the stimulation and oscillation frequency are matched (Holt et al., 2019). This demonstrates that using stimulation at the target frequency can allow the closed-loop amplifying or suppressing phases to be estimated without systematically searching the parameter space, as we have done here and in previous studies (McNamara et al., 2022). This has important implications for future clinical studies where in may be advantageous to find the suppressing or amplifying phase quickly.

There is precedent for using DBS to target features of an ongoing oscillation to improve symptom severity in diseases like Parkinson’s, where beta activity is pathologically synchronised (Little and Brown, 2014). Targeting 130Hz DBS to windows of increased beta power was found to both reduce the amplitude of beta oscillations and reduce symptom severity in those with Parkinson’s disease, outperforming continuous stimulation (Little et al., 2013). Equally, amplification of beta activity by beta-locked DBS has been found to worsen symptoms in parkinsonian rats (McNamara et al., 2022). The impact of AD on theta oscillations is not yet well-defined, with studies in animal models (Colom et al., 2010; Hu et al., 2018; Mahar et al., 2017; Mehak et al., 2022; Mondragón-Rodríguez et al., 2018; Scott et al., 2016; Siwek et al., 2015; Stoiljkovic et al., 2016; Villette et al., 2010; Wang et al., 2020) and humans (Caravaglios et al., 2010; Cummins et al., 2008; Hsiao et al., 2013; Ishii et al., 2017; Jafari et al., 2020; Luppi et al., 2022; Peña-Ortega and Bernal-Pedraza, 2012) showing variable relationships. Here we have demonstrated the potential of closed-loop phase-targeted fx-DBS to modulate theta in either direction, demonstrating that this approach can be readily adapted to pathophysiological changes in theta oscillations as they are identified in humans and novel animal models. Optogenetic stimulation phase-locked to the hippocampal theta oscillation has already been found to alter memory performance (Gava et al., 2024; Siegle and Wilson, 2014). Here we provide evidence that fx-DBS has the potential to achieve the same effects. As different target phases can be used to move the frequency and phase of theta oscillations in either direction, our approach may be used both to reduce pathophysiological, or restore normative oscillatory dynamics. Phase-targeted fx-DBS thus provides a clinically tractable approach for manipulating theta oscillations with the aim of improving memory performance in clinical populations.

## Supplementary Material

Supplementary Material
