# Supplementary material for: Phase-dependent closed-loop deep brain stimulation of the fornix provides bidirectional manipulation of hippocampal theta oscillations
Source: Brain Stimul. Author manuscript; Available in PMC 2025 Aug 19. (PMC7618027; doi:10.1016/j.brs.2025.04.019)
Supplement: Supplementary Material [file EMS207926-supplement-Supplementary_Material.pdf]

Supplemental methods

Surgical procedures

For implantation of the multi-electrode device, isoflurane anaesthesia (4% for induction, 1.5-2.5% maintenance) and oxygen (2L/min) were used. At the start of surgery, local anaesthetic (Marcaine, 2mg/kg, 2.5 mg/mL) and a non-steroidal anti-inflammatory drug (Meloxicam, 1mg/kg, 5mg/mL) were administered subcutaneously. For post-operative analgesia, an opioid (Buprenorphine, 0.3 mg/mL, 0.03 mg/kg) was given subcutaneously for three consecutive days.

A stereotaxic arm was used to mark the outer boundaries of craniotomy windows (David Kopf Instruments). The coordinates of these craniotomy windows were planned using the 4th and 6th versions of Paxinos and Watson's rat brain atlas (Paxinos and Watson, 2009). Craniotomies were then drilled above targeted brain structures and the underlying dura mater was removed. The drive was then aligned to the centre point of the craniotomy window above the fornix using the stimulation wire as a reference. The drive was then lowered to the required depth from the brain surface using a stereotactic arm. Bone cement was used to adhere the device to screws drilled into the skull.

Multi-electrode microdrive

A driveable multielectrode implant was used to record field potentials and single unit activity. These bespoke microdrives allow clusters of tetrodes (4 microwires) to be

independently moved through brain tissue by turning a screw. This device consisted of three main parts, a lid, a midsection and a base. The lid contained four blocks of 34 pin Mill-Max connectors; these interfaced the microwires to the recording headstages via a pushfit pin system. The midsection contained the screws, shuttles and guide rails to move the tetrodes. The base had holes drilled into it through which polyimide glass tubing containing tetrodes was fed. These devices were bespoke and built entirely in house (for further details of the building process see Härmson et al. 2023 (Härmson et al., 2023)). Tetrodes were produced by twisting 4 strands of coated 12.5-mircron diameter tungsten wire (California Fine Wire) together and then fusing the coating with a heat gun. For electrophysiological recordings, this device was then plugged into two 64-channel Intan head-stages (Intan Technology) via the Mill-Max connectors in the lid.

Devices contained 31 tetrodes, 2 ECOG screws and 2 supra-cerebellar screws which were used to ground electrophysiological recordings. In 3 of 5 animals 21 tetrodes were targeted to the cell layer of CA1 and 9 to the dorsal subiculum of the right hippocampus. In 2 of the 5 animals, all 31 tetrodes were targeted to the cell layer of CA1 of the right hippocampus. Only data recorded in CA1 of the hippocampus is analysed here. In all animals, the DBS electrode was targeted to the right fornix.

Tetrodes were lowered into the pyramidal cell layer of CA1 at the start of each recording day 1.5 – 2 hours prior to recording, and then raised back up 150um at the end of the day. This procedure was used to preserve the cell layer in CA1 and facilitate the identification of distinct single units.

Prior to and following linear track exploration, electrophysiological recordings were also made in a square open field enclosure and/or a sleep box. No stimulation was ever delivered outside of the linear track.

Fornix stimulation

Electrical stimulation was delivered from two fused strands of stainless-steel wire (insulated apart from the tip) in the fornix (Fig. S1), which together made up the stimulation wire, one of which acted as a current source and the other as a current sink. Both of these wires were 127μm in diameter and were separated by approximately 40μm. Each of these wires was connected to a fully isolated current source (A385, stimulus isolator, World Precision Instruments). The electrical stimulation was delivered biphasically at 50-100 μA and lasted 200μs. That is, an electrical stimulation was made up of two 95μs pulses of opposite polarity separated by 10μs.

Position and speed analysis

The position of the head was determined using a DeepLabCut model (Mathis et al., 2018) trained on 10 manually labelled frames per video from 246 videos spanning all 5 rats and all 3 stimulation types (phase-locked fx-DBS, replayed-fx-DBS, *no*-fx-DBS). Frames were extracted for labelling using the k-means algorithm, that clustered frames with similar visual appearances. Frames were then extracted from across multiple clusters to ensure that the labelled frames spanned multiple distinct behavioural states. The Resnet 50 model was then trained on these labelled frames. The model

was trained over 1030000 iterations until the loss had plateaued. Videos labelled by the resulting model were then visually inspected to validate model performance.

Given this experiment was conducted on a linear track, we considered the position of the animal to be a 1-dimensional timeseries, with this position indicating how far along the linear track the animal was. The instantaneous speed of animals was calculated as the absolute change in position (in cm) of the animal between frames of the video, multiplied by the sample rate of the video. Rats were classified as running if their instantaneous speed was greater than 20cm/s.

LFP analysis

All analysis was conducted on the LFP electrode that the OscillTrack algorithm used for determining the phase of oscillations online in real time. The stimulation artefact from DBS generally led to contamination of only a short window of time. As such, we found the artefact was effectively removed by interpolating between 0.2ms before and 0.6ms after the stimulation was delivered in the 20kHz wideband signal. For closed-loop stimulation and open-loop stimulation, the artefact removal process was conducted around each DBS pulse. In the *no*-fx-DBS condition, the artefact removal was carried out when the OscillTrack would have delivered stimulation if it was connected to the stimulation electrode. The *no*-fx-DBS condition, therefore, controls for any effects that would result from the artefact removal alone. The signal was then down sampled to 1000Hz using finite impulse response anti-aliasing filters from McNamara et al., 2022.

Welch's method was used to compute estimates of the power spectral density of the 1000Hz LFP signal using a segment length of 2^{12}. These were computed separately for each of the 3 DBS conditions that made up a stimulation day. For each power spectral density, the area under the curve, maximum value and frequency of maximum value in the 6-10Hz frequency range was computed. The change in each of these values in the phase-locked DBS condition from the *no*-fx-DBS condition was compared across the different phases of stimulation. Equally for each phase, comparisons were made between phase-locked DBS and the 2 control conditions (*no*-fx-DBS and replayed-fx-DBS). This was particularly to ensure that the changes in the power spectral density arose as a result of phase-locking, not the temporal pattern of the stimulation train (which was controlled for by the replayed-fx-DBS condition).

For the offline computation of phase and instantaneous amplitude, the LFP was bandpass filtered between 6 and 10Hz using a zero-phase lag 4th order Butterworth filter. The instantaneous phase and amplitude were computed from the Hilbert transform of this signal. The phase estimates computed were used to determine the phase that fornix DBS was delivered at. Triggered averages of the instantaneous amplitude of replayed-fx-DBS are shown in fig. 5, separating by the phase of the theta oscillation that DBS pulses coincided with. To compute the relationship between speed and theta power, the instantaneous amplitude of theta oscillations was averaged across the time between the adjacent frames of the video that were used to calculate the speed of the animal. This averaged amplitude of theta oscillations was then meaned separating by the speed of the animal in 5cm/s intervals.

Time-frequency power spectra were computed with repeated consecutive Fourier transforms. This was calculated in LFP with a sample rate of 1000Hz using a segment

length of 2^{10} and Hann windowing. The overlap was set to one less than the segment length to give maximal smoothing in the time domain. The spectra were then log transformed using the natural logarithm. For each session, we then computer the average spectrogram around the transition from slow (< 20cm/s) to fast (>20cm/s) movement. To test whether phase-dependent stimulation significantly affected the power across oscillatory bands we applied cluster-based permutation testing as per Maris and Oostenveld, 2007 (Maris and Oostenveld, 2007). We used the f-statistic from ANOVA for identification of clusters and then significance testing. This test statistic reflects the difference in power across the 4 phases of stimulation at a given frequency and time from speed transition. Adjacent times and frequencies with an f-statistic above a threshold corresponding to a p-value of 0.01 were grouped together into clusters. The cluster-level statistic was then calculated by taking the sum of f-statistics across the cluster. The largest cluster statistic repeating this procedure 1000 times with the phase-labels randomly permuted was then stored. If a cluster had a greater statistic than the 97.5th percentile of the permuted distribution, it was classified as significant.

Supplemental figures

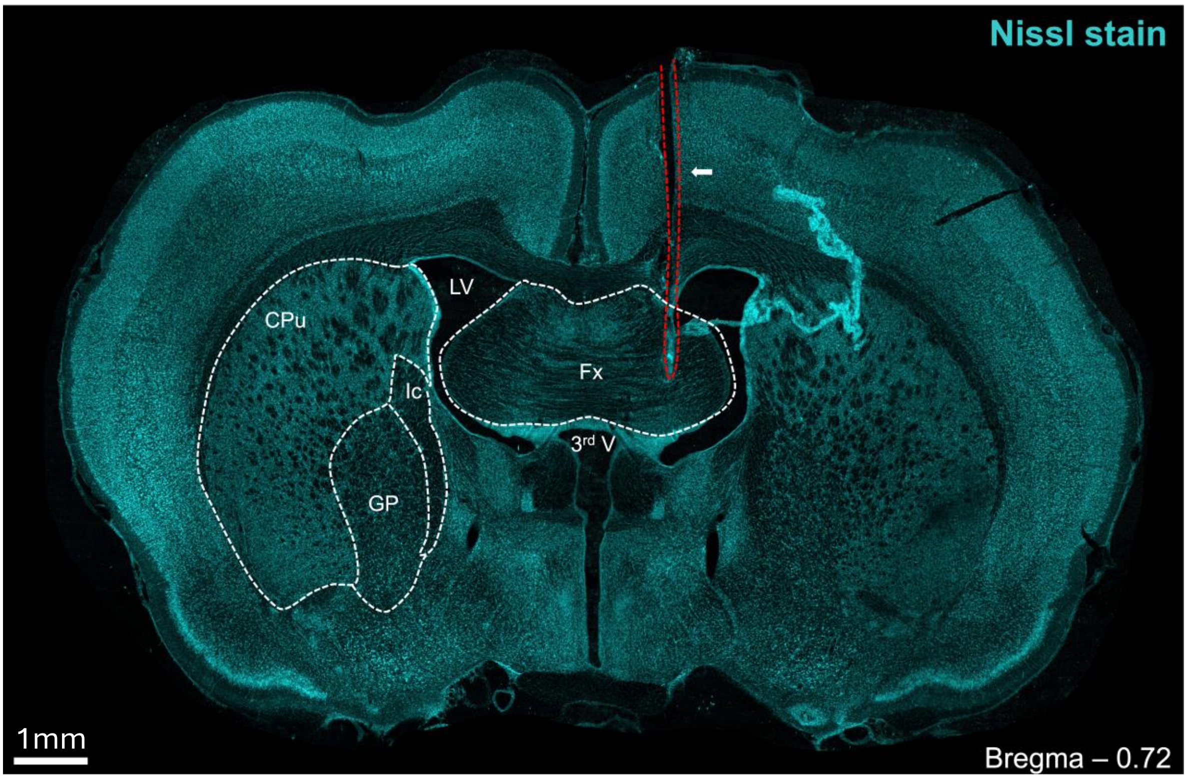

Figure S1. A coronal section from an implanted rat showing the position of the fornix DBS electrode. The electrode tract of the stimulation electrode into the right fornix is marked with a red dotted line. The borders between anatomical structures are marked with a white dotted line. Fx = fornix, LV = lateral ventricle, CPu = caudate putamen, GP = globus pallidus, 3rd V = third ventricle, Ic = Internal capsule.

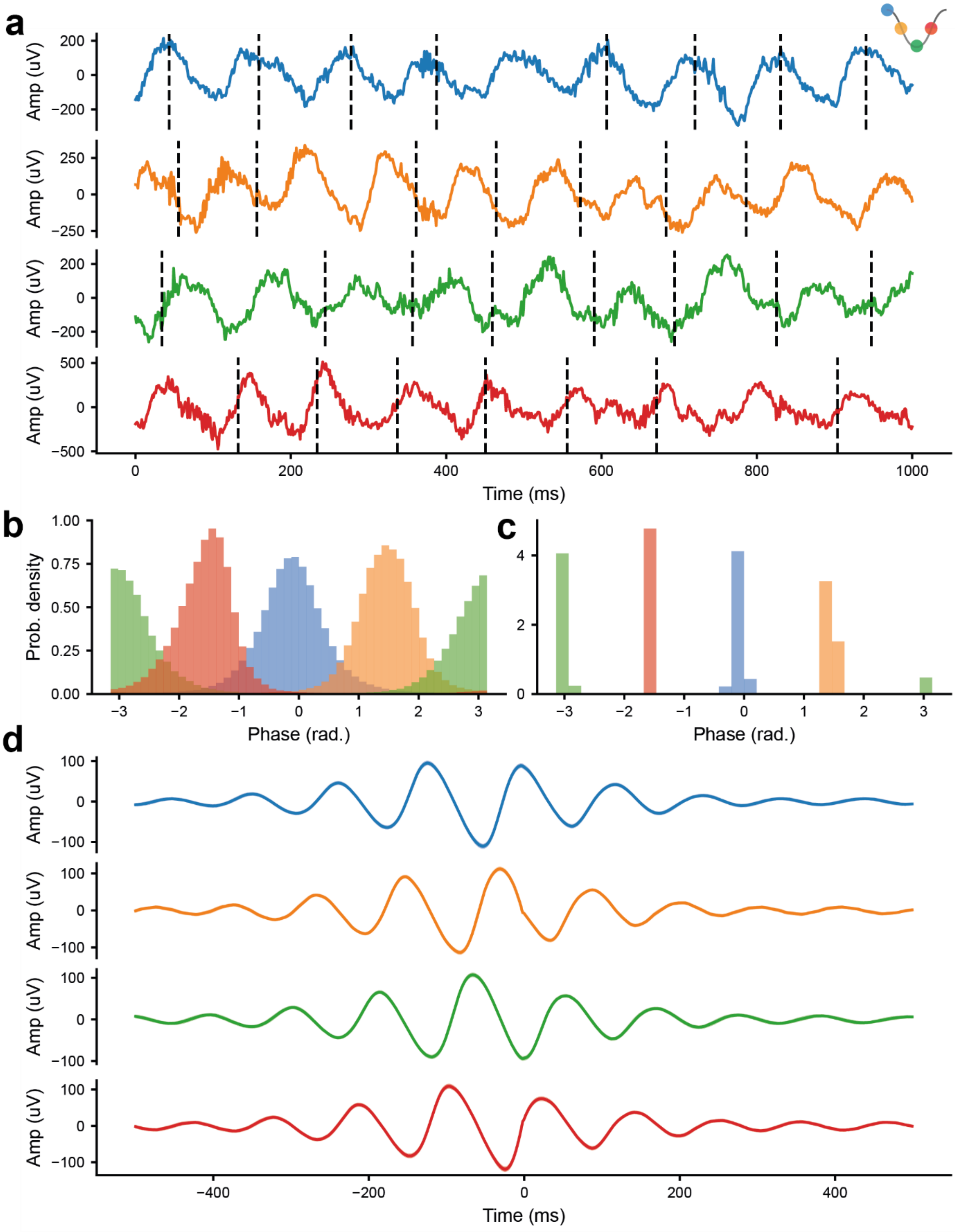

Figure S2. The OscillTrack algorithm was also able to track the phase of theta oscillations in CA1 of the hippocampus in the absence of fx-DBS: For all figure panels, blue=peak-phase, orange=descending-phase, green=trough-phase and red=ascending-phase tracking (see key in top right corner). Data presented here is from 5 rats over 83 recordings (peak-phase n=22, descending-phase n=22, trough n=20, ascending n=19). During *no*-fx-DBS stages, the OscillTrack algorithm was left on but was disconnected from the stimulation electrode. This allowed us to detect when the Osciltrack algorithm *would* have delivered stimulation during the *no*-fx-DBS stages. **a)** Figure shows the

wideband LFP. The times where stimulation would have been delivered are marked with a vertical dashed line. **b and c)** The wideband LFP was filtered in the theta band (6-10Hz), and the phase that pulses would have been delivered during *no*-fx-DBS stages was determined offline using the Hilbert transform. **b)** The phase that fx-DBS would have been delivered at pooled across all *no*-fx-DBS stages of each type of phase tracking (peak, descending, trough and ascending phases). **c)** The circular mean of the phase at which fx-DBS pulses would have been delivered for each *no*-fx-DBS stage separating by the phase tracking type (peak, descending, trough and ascending phases). **d)** The average of wideband LFP triggered by when fx-DBS pulses would have been delivered in *no*-fx-DBS stages, averaged across recording stages for each of the four tracking phases.

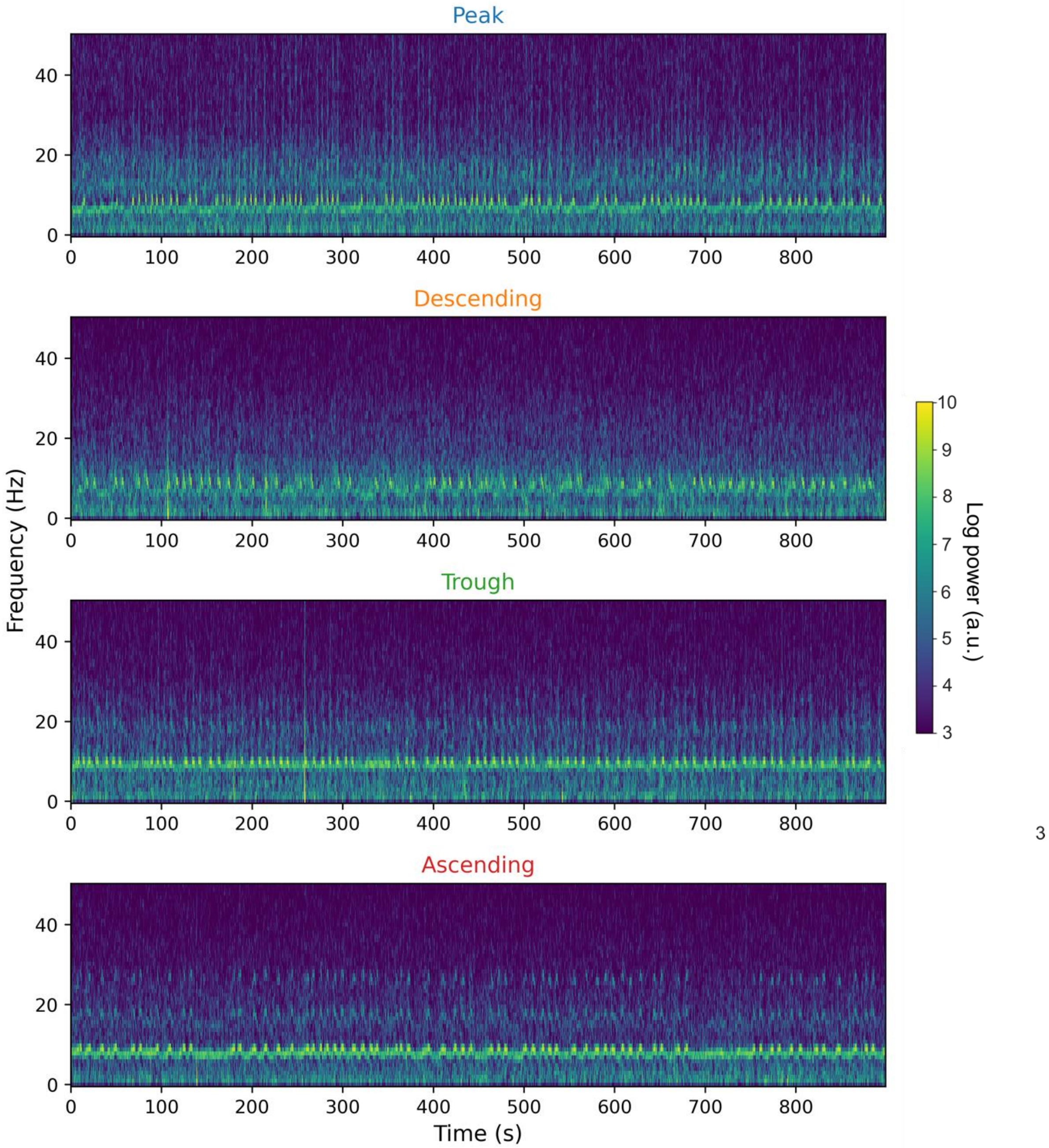

Figure S3. A strong and stable theta oscillation was present across recording sessions: The log time-frequency spectra of the hippocampal LFP in 4 example recordings. All 4 were from phase-locked fx-DBS stages in a single animal. From top to bottom, the log time-frequency spectra were from peak-, trough-, descending- and ascending-locked stimulation (see labels above each spectrum).

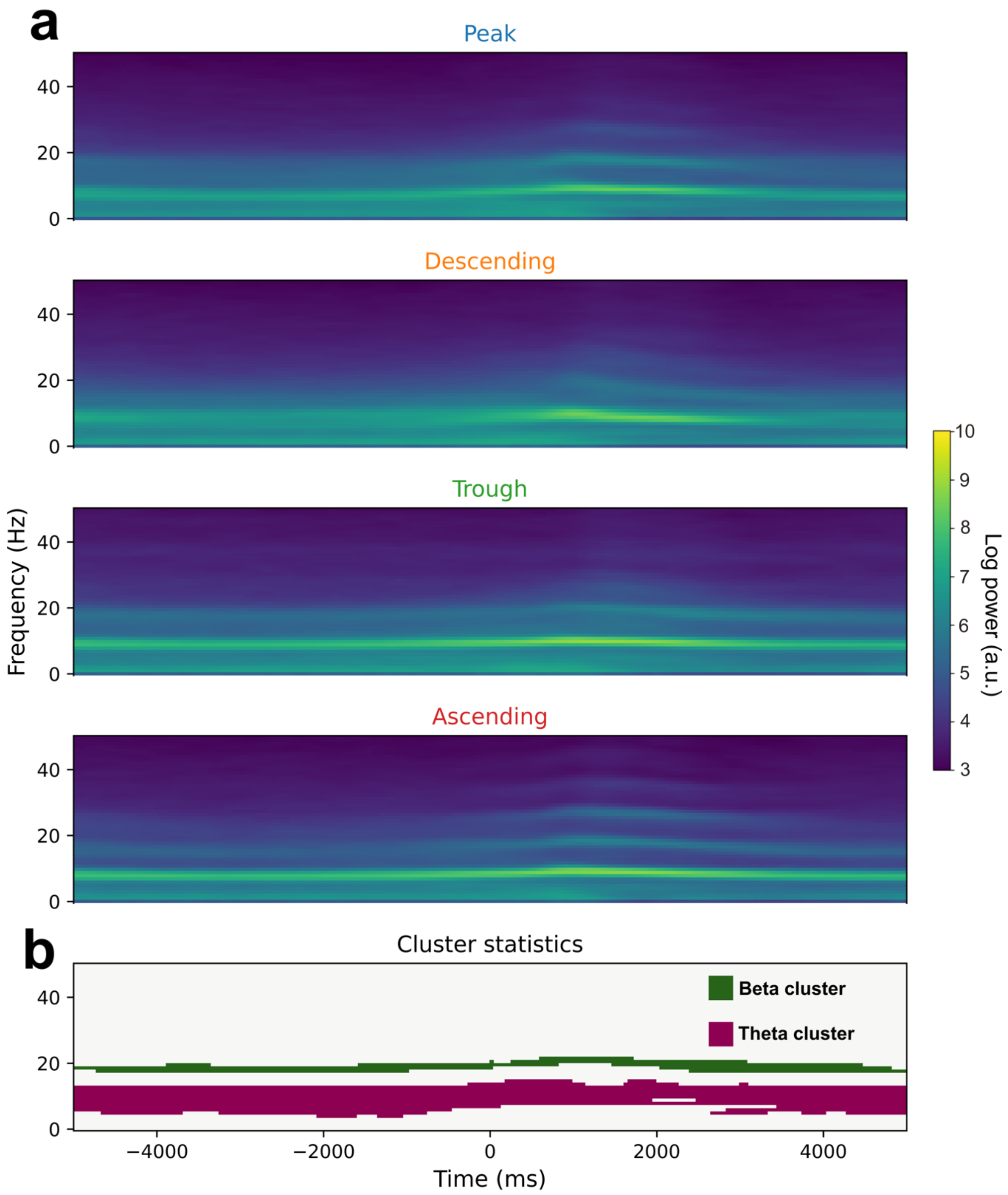

Figure S4. Phase-locked fx-DBS modulated theta power across a range of behavioural states: a) The triggered average of the log time-frequency spectra for each of the 4 phases of phase-locked fx-DBS, averaging around the transition from slow movement (i.e., <20cm/s on average over 30 frames) to fast movement (i.e., >20cm/s on average over 30 frames). From top to bottom, the averaged log time-frequency spectra were plotted for peak-, descending-, trough- and ascending-locked stimulation (see labels above each spectrum). Time 0ms represents the transition from slow to fast movement. The spectrogram was aligned with the positional data such that the spectrogram at time 0ms represents the power of oscillations in the LFP 1024ms prior to the speed transition. **b)** Significant time-frequency clusters were determined using cluster-based permutation statistics. Clusters were identified by finding adjacent time/frequency points where the oscillatory power significantly differed (p<0.01) across the different target-phases of stimulation. The f-statistic from the ANOVA (comparing oscillatory power at a

given time/frequency point across the different target-phases of stimulation, as above) was then summed across the time/frequencies within the cluster. A cluster was classified as significant if its summed f-statistic was above the 97.5th percentile of a null distribution produced by randomly permuting the phase-labels (see methods). Two significant clusters were identified, one in the theta band (purple) and one in the low-beta (approximately 17-22Hz) band (green). Given that the low-beta cluster is at roughly twice the frequency of the theta-band, the modulation of the beta oscillations observed here likely reflect changes in the harmonics of the prominently observed theta oscillation.

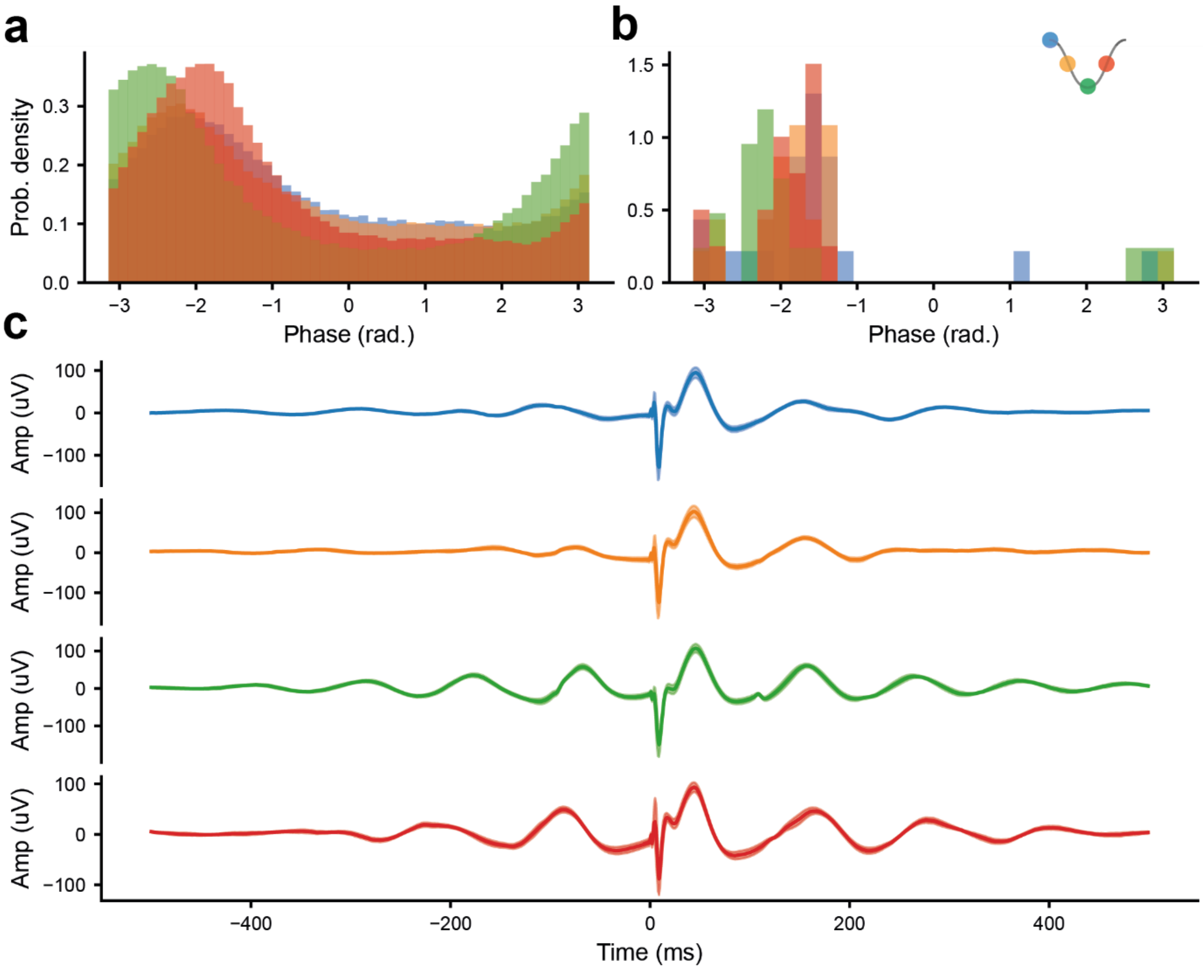

Figure S5. Replayed-fx-DBS had a tendency to entrain theta oscillations but had no algorithmically determined relationship to theta-phase: For all figure panels, blue=peak-phase, orange=descending-phase, green=trough-phase and red=ascending-phase (see key in top right corner). Data presented here is from 5 rats over 83 recordings (peak-phase n=22, descending-phase n=22, trough n=20, ascending n=19). The wideband LFP was filtered in the theta band (6-10Hz), and the phase that fx-DBS pulses occurred at was determined offline using the Hilbert transform. **a)** The phase of all of DBS pulses pooled across all replayed-fx-DBS stages of each type (peak, descending, trough and ascending phases). **b)** The circular mean of the phase of DBS pulses for each replayed-fx-DBS stage separating by the phase of replayed-fx-DBS (peak, descending, trough and ascending phases). **c)** The average of wideband LFP triggered by replayed-fx-DBS pulses, averaged across recording stages by which of the four phases was being replayed.
